# Nociceptor Sensitization Depends on Age and Pain Chronicity^1^,^2^,^3^

**DOI:** 10.1523/ENEURO.0115-15.2015

**Published:** 2016-02-08

**Authors:** Andy D. Weyer, Katherine J. Zappia, Sheldon R. Garrison, Crystal L. O’Hara, Amanda K. Dodge, Cheryl L. Stucky

**Affiliations:** 1 Department of Cell Biology, Neurobiology, and Anatomy, Medical College of Wisconsin, Milwaukee, Wisconsin 53226; 2 Promentis Pharmaceuticals, Milwaukee, Wisconsin 53203

**Keywords:** acute pain, aging, chronic pain, mechanical, sensitization, skin–nerve preparation

## Abstract

Peripheral inflammation causes mechanical pain behavior and increased action potential firing. However, most studies examine inflammatory pain at acute, rather than chronic time points, despite the greater burden of chronic pain on patient populations, especially aged individuals. Furthermore, there is disagreement in the field about whether primary afferents contribute to chronic pain. Therefore, we sought to evaluate the contribution of nociceptor activity to the generation of pain behaviors during the acute and chronic phases of inflammation in both young and aged mice. We found that both young (2 months old) and aged (>18 months old) mice exhibited prominent pain behaviors during both acute (2 day) and chronic (8 week) inflammation. However, young mice exhibited greater behavioral sensitization to mechanical stimuli than their aged counterparts. Teased fiber recordings in young animals revealed a twofold mechanical sensitization in C fibers during acute inflammation, but an unexpected twofold reduction in firing during chronic inflammation. Responsiveness to capsaicin and mechanical responsiveness of A-mechanonociceptor (AM) fibers were also reduced chronically. Importantly, this lack of sensitization in afferent firing during chronic inflammation occurred even as these inflamed mice exhibited continued behavioral sensitization. Interestingly, C fibers from inflamed aged animals showed no change in mechanical firing compared with controls during either the acute or chronic inflammatory phases, despite strong behavioral sensitization to mechanical stimuli at these time points. These results reveal the following two important findings: (1) nociceptor sensitization to mechanical stimulation depends on age and the chronicity of injury; and (2) maintenance of chronic inflammatory pain does not rely on enhanced peripheral drive.

## Significance Statement

Most peripheral pain research examines acute pain in young animals, with the assumption that peripheral pain mechanisms are similar during acute pain and chronic pain for animals of all ages. Our results indicate that peripheral nociceptors may contribute minimally to pain sensation at chronic inflammatory time points in young populations, and at either acute or chronic time points in aged populations. These findings have important implications for novel analgesic design, as drugs targeting peripheral pain mechanisms observed under acute inflammatory conditions may be unlikely to show efficacy under chronic inflammatory conditions. Additionally, since nociceptors from aged animals do not change their firing rates in response to acute or chronic pain, peripherally acting analgesics may also be largely ineffective in aged populations.

## Introduction

Chronic pain results in hundreds of billions of dollars in economic costs in the United States (Committee on Advancing Pain Research, Care, and Education, 2011), but, despite a massive research effort over the past few decades, the successful translation of novel analgesics from preclinical models to the clinic has dwindled (Percie du Sert and Rice, 2014). While the cause of this drought is multifactorial, one of the primary sources may be limitations in the animal models used to elucidate the mechanisms of pain at the molecular level (Berge, 2011). Specifically, a significant shortcoming for many pain models, especially those examining inflammatory pain, has been the brief time course over which pain behaviors and molecular mechanisms are examined (Berge, 2011). Because of pressures related to animal housing costs, planning, and time to complete experiments, most studies involving inflammatory pain examine relatively acute time points following injury instead of true chronic time points that are often most relevant clinically (Wilson et al., 2006; Berge, 2011).

As a result, researchers have long inferred that the mechanisms discovered during the acute inflammatory pain phase remain constant even as pain becomes chronic, and that any drug targets identified acutely will also be reliable targets chronically. However, this premise has rarely been tested in animal models of bona fide chronic inflammatory pain (Wilson et al., 2006).

As an extension of this uncertainty, there is long-standing disagreement in the field over whether chronic pain is mediated by a combination of peripheral (primary afferent) and central (spinal cord/brain) mechanisms, or just by central mechanisms alone. However, because few studies have mechanistically examined pain sensation during chronic time points, this question is still unresolved. This is an important concern, as much research has focused on identifying potential drug targets in the peripheral nervous system in an effort to combat chronic pain (Cairns, 2009).

Although chronic pain affects individuals of all ages, one group it affects disproportionately is the elderly. Recent health surveys have found that >50% of individuals over the age of 65 years have complaints of pain, and that in 30% of these patients the pain is bad enough to interfere with the completion of activities of daily living (Thomas et al., 2004; Mottram et al., 2008; Patel et al., 2013, 2014). This pain is the result of a variety of pathologies that involve inflammatory mechanisms, including rheumatoid arthritis, osteoarthritis, gout, and musculoskeletal pain (Bruckenthal et al., 2009). However, a common thread among all of these is that the pain experienced by aged patients is often unresolved despite pharmacological treatment (Cavalieri, 2005; Tracy and Sean Morrison, 2013). Although this is becoming recognized as a considerable problem at the clinical level, comparatively little basic research has been conducted on pain mechanisms in aged animal models, and those studies that have examined pain responses in aged animals have shown conflicting results (Yezierski, 2012).

Therefore, using a mouse model of truly chronic inflammatory pain, we sought to determine whether mechanical pain sensation changes with age, and, furthermore, whether the peripheral nervous system contributes to mechanical pain sensation at chronic time points in both young and aged animals. Using a combination of behavioral, electrophysiological, and molecular approaches, here we show that age affects pain sensation under both basal and chronic inflammatory conditions, and, surprisingly, that peripheral afferent drive contributes minimally to the behavioral sensitization during the chronic phase of an inflammatory injury.

## Materials and Methods

### Animals

“Young” mice were 7–20 weeks of age ($\bar{x}$ = 13.6 ± 0.69 weeks) at the start of behavioral testing (and thus 15–28 weeks of age at the time of electrophysiological experiments). “Aged” mice were all >77 weeks of age ($\bar{x}$ = 94.4 ± 1.1 weeks) at the start of behavioral testing (85–108 weeks at the time of electrophysiological experiments). Mice that are 20 weeks of age correspond approximately to a 27-year-old human, while mice that are 100 weeks of age correspond approximately to a 67-year-old human (Flurkey et al., 2007). Animals used in these experiments were all male. Mice were predominantly from a mixed C57BL/6/outbred Swiss Webster/CBA background (https://www.jax.org/strain/004782); three aged animals were from a C57BL/6-only background, but no differences were observed between these animals and the mixed background animals. Animals were housed in a climate-controlled room with a 14 h:10 h light/dark cycle and *ad libitum* access to food and water. All behavioral assays and research protocols involving animals were approved by the Institutional Animal Care and Use Committee at the Medical College of Wisconsin.

### Behavior

Behavioral testing for mechanical sensitivity was performed in a dedicated behavioral suite at the Medical College of Wisconsin. Prior to testing, animals were placed in a small plastic chamber situated on a wire mesh that allowed access to mechanical probing of the plantar paw. Animals were habituated in these chambers for at least 1 h prior to testing. After the habituation period, the experimenter used calibrated von Frey filaments (North Coast Medical) to mechanically stimulate the glabrous skin of the hindpaw. The Up–Down method was used to determine paw withdrawal thresholds, as described previously (Chaplan et al., 1994). Additionally, a repeated, suprathreshold 3.61 mN von Frey filament was applied to the hindpaw 10 times, and the number of responses to this stimulus were recorded. For both the Up-Down test and the suprathreshold test, sufficient time was given between each stimulus to avoid sensitization of the paw.

For the capsaicin behavioral tests, mice were habituated in a small cage on a wire mesh for at least 30 min. Animals were then lightly anesthetized with isoflurane, and 30 μl of 100 μm capsaicin dissolved in 1% 1-methyl-2-pyrrolidone was injected into the left hindpaw. Animals were then videotaped for 5 min, and the number of licking/biting behaviors during this time were then analyzed. Blinding was not possible for these experiments as a result of the significant swelling observed in animals injected with complete Freund's adjuvant (CFA).

### Inflammation induction

Following basal mechanical sensation testing, young or aged mice were lightly anesthetized via inhaled isoflurane and injected subcutaneously with 30 μl of either sterile phosphate-buffered saline (PBS) or CFA into the left hindpaw. CFA injection resulted in a significant circumferential swelling of the hindpaw coupled with redness and decreased weight bearing that was visually observable. Signs and symptoms of inflammation were noticeable for the duration of the study (at least 8 weeks after injection). We considered the acute inflammatory phase to last from injection of CFA through the first 2 weeks after injection, and the chronic inflammatory phase to include weeks 3–8 postinjection, in accord with previous studies examining the transition from acute to chronic pain (Schwartz et al., 2013; Garrison and Stucky, 2014).

### Histology

To obtain and examine immune infiltration of the whole paw, paws were fixed in 10% neutral buffered formalin. Specimens were then decalcified and embedded in paraffin blocks. Coronal sections were then made at the level of the metatarsal-phalangeal joint and were stained with hematoxylin and eosin (H&E) for histologic analysis.

### Paw metrics

At the time of death, a digital caliper (VWR) was used to measure the width of the affected paw across the metatarsal-phalangeal joints and the height from the plantar surface of the paw to the dorsal surface across the head of the third metatarsal.

### Teased fiber electrophysiology

To assess primary afferent firing, we used saphenous skin–nerve preparations, as described previously (Reeh, 1986). Briefly, animals were lightly anesthetized and then killed via cervical dislocation. The leg was then quickly shaved with commercial clippers, and the hairy skin and innervating saphenous nerve were quickly removed from the carcass and placed in a heated (32 ± 0.5°C), oxygenated bath consisting of the following (in mm): 123 NaCl, 3.5 KCl, 0.7 MgSO_4_, 1.7 NaH_2_PO_4_, 2.0 CaCl_2_, 9.5 sodium gluconate, 5.5 glucose, 7.5 sucrose, and 10 HEPES. The buffer in the bath was titrated to a pH of 7.45 ± 0.05. The skin was then pinned down and the saphenous nerve was placed in a mineral oil-filled chamber and teased into small fascicles. Nerve bundles were then placed on the recording electrode, and a blunt glass probe was used to mechanically stimulate the preparation to identify single-unit receptive fields. C fibers displayed conduction velocities of <1.2 m/s, and A-mechanonociceptors (AMs) displayed conduction velocities between 1.2 and 10 m/s (Koltzenburg et al., 1997). All fibers used for these experiments exhibited slow adaptation to a sustained mechanical stimulus

Once identified, the basal activity of each fiber was recorded for 30-120 s. A feedback-controlled mechanical simulation device was then placed over the receptive field, and an increasing series of 15, 35, 70, and 140 mN forces was applied to the receptive field for 12 s each. A 1 min interval was given between each mechanical stimulus to prevent sensitization/desensitization of the fiber.

For another set of experiments, the responsiveness of C fibers to capsaicin was tested. Once the receptive field of a C fiber was identified, a metal ring was sealed around the receptive field using vacuum seal grease. Baseline recordings were then made for 2 min to establish a basal firing rate. The buffer within the metal ring was then evacuated and replaced with a solution containing 10 μm capsaicin dissolved in 0.1% 1-methyl-2-pyrrolidone for 2 min. Recordings were then analyzed off-line, and action potentials fired at baseline were subtracted from action potentials fired during capsaicin incubation. To be considered a “responder” to capsaicin, we required that a fiber fire a net of three action potentials over the duration of the 2 min incubation.

### Quantitative real-time PCR

Quantitative real-time PCR (qRT-PCR) was performed on L2–L5 dorsal root ganglia (DRGs) taken from experimental animals at the time of death. Samples were stored in RNALater solution at −20°C until the time of extraction. DRG samples were first manually homogenized in Trizol (Life Technologies), and RNA was then extracted using the Purelink RNA Micro Scale Kit (Life Technologies). RNA samples were then reverse transcribed into cDNA using the Superscript Variable Input Linear Output cDNA Synthesis Kit (Life Technologies). qRT-PCR was performed on a Mastercycler ep Realplex^2^ thermal cycler (Eppendorf) using TaqMan primers (Life Technologies) according to the manufacturer instructions. Context sequences and assay identifications (IDs) can be found in Table 1. Three technical replicates were averaged to obtain a mean cycle time for a given transcript.

**Table 1:** Context sequences for primers used for qRT-PCR

**Gene**	**TaqMan assay ID**	**Context sequence**
*scn9a*	Mm00450762_s1	ACGAAAGCAGGAAATAGAGCTTCGG
*scn10a*	Mm00501467_m1	TCCACTCCTGGTTCTCCATATTTAT
*scn11a*	Mm00449367_m1	TCTGTAATCTCAGGTCTGAAGGTCA
*fam38b*	Mm01265861_m1	ACAAGAGCCTCTTGTGCAAGAGGAG
*trpa1*	Mm01227437_m1	GAAGAAGGGAACACAGCACTCCACT
*trpv1*	Mm01246302_m1	TACTTTTCTTTGTACAGTCACTGTT
*trpc3*	Mm00444690_m1	CCTTGTAGCAGGCTGGGGAAGATTC
*trpc6*	Mm01176083_m1	TACCCCAGCTTCCGGGGTAATGAAA
*kcna1*	Mm00439977_s1	TGCGGCCGCACGCTCCCTGCCCCAC
*kcnq2*	Mm00440080_m1	CCACGCCTACGTGTTCCTTTTAGTC
*kcnq3*	Mm00548884_m1	TGTGCCCACAGCAAAGAACTCATCA
*tbp*	Mm00446971_m1	TCCCCACAGGGCGCCATGACTCCTG

### Data analysis and statistics

All statistical tests were performed using Prism software (version 5, GraphPad Software). For behavioral testing, paw withdrawal thresholds and the percentage of responses were compared between groups over time using a two-way repeated-measures ANOVA with Bonferroni *post hoc* test for significance at individual time points. Comparisons of basal (prior to injection) mechanical sensitivity were made using a nonparametric Mann–Whitney test. Capsaicin behavior was compared between groups using a one-way ANOVA.

For skin–nerve recordings, data were digitized using a PowerLab analog-to-digital converter (AD Instruments) and analyzed off-line using LabChart 7 Software with the Spike Histogram extension (AD Instruments). Recordings were only used if the recorded fiber was clearly distinguishable by action potential profile from background noise and other fibers firing during the mechanical stimulation. Comparisons between groups over the force series were made using a two-way ANOVA with Bonferroni *post hoc* analysis. von Frey thresholds of individual C fibers were compared between CFA- and PBS-injected groups using a nonparametric Kruskal–Wallis test. Spontaneous firing of C fibers was performed using a contingency table with Fisher’s exact test. Binned interspike intervals (ISIs) were compared through the use of a χ^2^ with Fisher’s exact test. Coefficients of Variation (CV_2_) were determined by the following equation: ((√2)*σ)/$\bar{x}$, where σ is the SD of two adjacent ISIs, and $\bar{x}$ is the average of those two ISIs (Holt et al., 1996). All CV_2_ for a given spike train were then averaged to yield a single number that was compared between cohorts using a one-way ANOVA with a Bonferroni *post hoc* analysis for specific comparisons. The percentage of responders to capsaicin was compared using a χ^2^ test followed by Fisher’s exact test. The number of action potentials fired in response to capsaicin incubation was compared using a one-way ANOVA.

For qRT-PCR, the change in cycle time between the gene of interest and the control gene was compared between PBS-injected and CFA-injected groups using a Student’s *t* test to determine significant changes in gene expression at a given time point for a specific group. Changes between groups were analyzed using a one-way ANOVA of the fold changes for each group with Bonferroni *post hoc* analysis.

Prior to study initiation, we set the α level to *p* = 0.05. All statistical tests utilized in this study are summarized in Table 2, which can be found at the conclusion of this manuscript.

## Results

### Young mice exhibit greater inflammatory mechanical sensitization than aged mice

**Figure 1.**
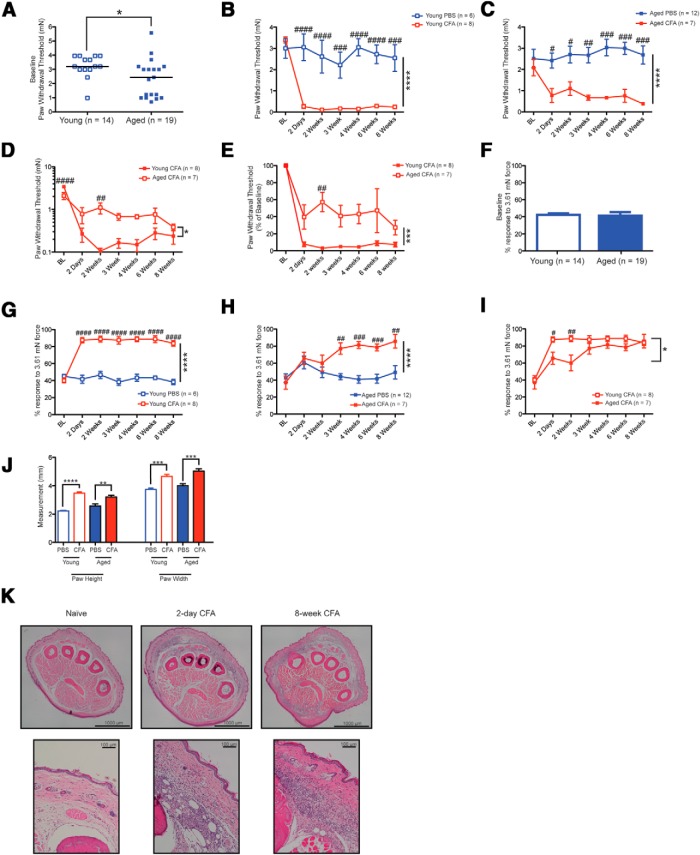
Acute and chronic inflammation sensitizes behavioral responses to mechanical stimuli to different extents in young and aged mice. ***A***, Paw withdrawal thresholds to a mechanical stimulus are lower in aged animals (2.35 mN) compared with young animals (3.23 mN) at baseline. ***B***, ***C***, Injection of CFA results in a dramatic reduction in paw withdrawal thresholds both acutely and chronically in young (***B***) and aged (***C***) mice compared with PBS injection. ***D***, Young mice exhibit a greater reduction in paw withdrawal thresholds compared with aged mice. ***E***, As a percentage of baseline, young mice exhibit a >90% reduction in paw withdrawal thresholds, while aged mice exhibit a 40-75% reduction in paw withdrawal thresholds. ***F***, Baseline responses to a 3.61 mN suprathreshold stimulus are similar between young and aged mice. ***G***, ***H***, Injection of CFA results in a significant elevation in the percentage of response to a suprathreshold 3.61 mN stimulus in both young (***G***) and aged (***H***) mice. ***I***, In response to the injection of CFA, aged mice respond with elevations in the percentage response to a suprathreshold stimulus on a different time course than young mice. ***J***, Chronically inflamed mice continue to exhibit significant paw swelling at 8 weeks after inflammation induction. ***K***, Top row, H&E-stained coronal sections through the entire paw at the metatarsophalangeal joint from young animals show significant inflammatory infiltrate present at both 2 days and 8 weeks after CFA injection. Bottom row, Increased magnification of the whole-paw sections demonstrate significant infiltration of neutrophils and monocytes/macrophages at both 2 days and 8 weeks of CFA-mediated inflammation.

Few studies have examined mechanical sensation in preclinical studies using aged rodents, and those that have offer discordant results: increased sensitivity in aged rats (Kitagawa et al., 2005), decreased sensitivity in aged mice (Garrison and Stucky, 2014) or no change between young and aged rats (Taguchi et al., 2010) have all been reported. Therefore, we first assessed whether age affects mechanical sensation by measuring paw withdrawal thresholds in young (13 weeks) and aged (>77 weeks) mice. We found that naïve aged mice exhibited lower mechanical thresholds than naïve young animals (mean, 2.35 vs 3.22 mN for young animals), indicating an elevated basal sensitivity to mechanical stimuli with older age (Fig. 1A ^a^; ^*^p < 0.05, Mann–Whitney test; *n* = 19 animals for aged group; *n* = 14 for young group).

Past studies examining changes in pain perception during aging have found discordant results, with about half of published reports indicating that aged animals have increased pain sensitivity, and the other half indicating that aged animals have diminished pain sensation or unaltered pain sensation compared to young animals (for review, see Yezierski, 2012). Therefore, we next considered the effect of a painful inflammatory insult on mechanical thresholds in these populations by injecting CFA subcutaneously into the plantar hindpaw. In comparison with mice injected with PBS, both young and aged animals injected with CFA showed a sharp decline in mechanical paw withdrawal thresholds from the acute inflammatory phase (2 day and 2 week time points) through the chronic inflammatory phase (3–8 week time points; Fig. 1B ^b^,*C* ^c^; ^****^p < 0.0001, two-way repeated-measures ANOVA; ^#^*p* < 0.05, ^##^*p* < 0.01, ^###^*p* < 0.001, and ^####^*p* < 0.0001, Bonferroni *post hoc* test for multiple comparisons; *n* = 6-12 animals as noted in Fig. 1B ^b^,*C* ^c^). Although young and aged mice both displayed significant reductions in paw withdrawal thresholds following inflammation induction, the amount of sensitization was markedly different between these two age groups. From the end of the acute phase (2 weeks) through much of the chronic phase, aged inflamed mice displayed mechanical thresholds that were 4- to 10-fold higher (less sensitive) than young mice (Fig. 1D ^d^; ^*^p < 0.05 using a repeated-measures two-way ANOVA; ^##^*p* < 0.01 and ^####^*p* < 0.0001 with Bonferroni *post hoc* test, *n* = 8 and 7 animals). Compared to their baseline mechanical thresholds, aged inflamed mice showed reductions in mechanical paw withdrawal thresholds between 43% and 73% over the duration of testing, while young mice showed 91–97% reductions in paw withdrawal thresholds over the same period (Fig. 1E ^e^; ^***^p < 0.001 with a two-way repeated-measures ANOVA; ^##^*p* < 0.01 with Bonferroni *post hoc* analysis; *n* = 8 and 7 animals).

We further examined the responses of young and aged mice to a repeated 3.61 mN von Frey filament in order to test mechanical responsiveness to suprathreshold stimuli. While a reduction in mechanical thresholds is characteristic of allodynia, increased responsiveness to a suprathreshold stimulus may be an indication of hyperalgesia. In contrast to the age differences observed for mechanical thresholds, response frequencies to a suprathreshold mechanical stimulus were not different at baseline between young and aged mice (Fig. 1F ^f^; *p* > 0.05, Student’s *t* test; *n* = 14 and 19 animals). Following inflammation induction, both young and aged mice exhibited significant elevations in response frequencies to the suprathreshold stimulus, with each group ultimately responding approximately 80% of the time compared with 40% at baseline (Fig. 1G ^g^,*H* ^h^; ^****^p < 0.0001, two-way repeated-measures ANOVA; ^#^*p* < 0.05, ^##^*p* < 0.01, ^###^*p* < 0.001, and ^####^*p* < 0.0001 with Bonferroni *post hoc* test for multiple comparisons; *n* = 6-12 animals as noted in Fig. 1G ^g^,*H* ^h^). Interestingly, however, the time course of the sensitization to suprathreshold stimuli was different between young and aged mice. Whereas young mice injected with CFA responded 80% of the time to a suprathreshold stimulus within 2 days of inflammation induction, aged mice injected with CFA exhibited responses to suprathreshold stimuli that were similar to those of controls until 3 weeks after injection, in conjunction with the beginning of the chronic phase of pain (Fig. 1I ^i^; ^*^p < 0.05 with two-way repeated-measures ANOVA; ^#^*p* < 0.05, ^##^*p* < 0.01 with Bonferroni *post hoc* test for multiple comparisons; *n* = 8 and 7 animals). This complements previous reports from both animal models of pain and human studies indicating that aged subjects may develop experimental pain more slowly than young participants (Zheng et al., 2000; Cruce et al., 2001).

Also of note is that in our hands, von Frey thresholds and suprathreshold response frequencies never returned to baseline throughout the 8 weeks following CFA injection and instead exhibited quite pronounced sensitization at 8 weeks. This matches our observations of significant swelling and redness in the injected paw, which continued to be present at least 8 weeks after the initial injection (Fig. 1J ^j^; ^**^p < 0.01, ^***^p < 0.001, ^****^p < 0.0001, one-way ANOVA with Bonferroni *post hoc* test; *n* = 7 animals for aged CFA group, *n* = 5 animals for aged PBS group, *n* = 5 animals for young CFA group, and *n* = 8 animals for young PBS group). Furthermore, H&E-stained paw sections from naïve, acutely inflamed, and chronically inflamed young animals demonstrate consistent infiltration of immune cells at both 2 days and 8 weeks after CFA injection in accord with a recent report (Ghasemlou et al., 2015; Fig. 1K). Behavioral testing of the contralateral (uninjected) paw yielded no differences in mechanical sensitivity compared with controls (data not shown).

Collectively, these data suggest that, although both young and aged animals display significant pain behaviors during long-standing inflammation, aged animals have a blunted response to inflammatory pain.

### Young, but not aged, C-fiber nociceptors are sensitized during acute inflammation

Since behavioral pain responses were notably different between young and aged animals, we next wondered whether this was reflected in the firing of primary afferents from these animals. The presence of peripheral sensitization to mechanical stimuli following acute inflammatory injuries has been debated, with some research indicating that primary afferents are sensitized to mechanical stimuli following inflammation (Andrew and Greenspan, 1999; Potenzieri et al., 2008; Lennertz et al., 2012; Smith et al., 2013), while other research does not show an elevation in nociceptive firing following peripheral injury (Kocher et al., 1987; Koerber et al., 2010; Schmidt et al., 2012). Although recent research has indicated that myelinated fibers may play an important role in mechanical hyperalgesia following CFA-mediated inflammation (Meyer et al., 1991; Andrew and Greenspan, 1999; Potenzieri et al., 2008; Weyer et al., 2015), we chose to first focus on unmyelinated C fibers, since this afferent class has traditionally been understood to transmit painful stimuli to the CNS.

**Figure 2.**
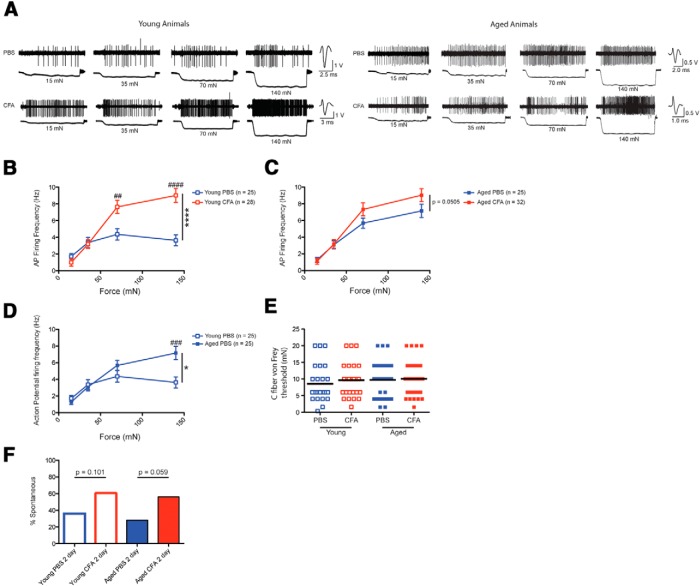
Acute inflammation sensitizes C-fiber nociceptors to mechanical force only in young animals. ***A***, Trace examples from young animals injected with either PBS (top left) or CFA (bottom left) and aged animals injected with either PBS (top right) or CFA (bottom right). ***B***, C-fiber nociceptors from acutely inflamed (2 day) young animals respond with significantly higher action potential firing rates in response to increasing mechanical forces. ***C***, C-fiber nociceptors from acutely inflamed aged animals trend toward responding with increased action potential firing in response to increasing mechanical forces, but this relationship is not significant. ***D***, At baseline, C fibers from aged animals are more sensitive to mechanical stimuli than C fibers from young animals. ***E***, von Frey thresholds for individual C fibers were not different between the four cohorts. Each point on the graph represents the von Frey threshold of an individual C fiber, and the black bars are indicative of the group mean. ***F***, More C fibers from acutely inflamed animals tend to have ongoing, nonevoked activity (>0.05 Hz), although this relationship is not significant.

We first examined the effect of acute inflammation on C fiber firing in young and aged animals using an *ex vivo* skin–nerve preparation (Fig. 2A). We noted a significant twofold sensitization in action potential firing to a series of increasing mechanical forces in C-fiber afferents from young animals when skin–nerve preparations were harvested 2 days after CFA injection (Fig. 2B ^k^; ^****^p < 0.0001 with two-way ANOVA, ^##^*p* < 0.01 and ^####^*p* < 0.0001 with Bonferroni *post hoc* test; *n* = 25 fibers for PBS and 28 fibers for CFA, data obtained from three animals in each group). In contrast, we found that C fibers from aged animals exhibited a strong trend toward sensitization to mechanical stimuli following acute CFA inflammation compared with PBS controls, but this relationship was not statistically significant (Fig. 2C ^l^; *p* = 0.0505 with two-way ANOVA; *n* = 25 fibers for PBS and *n* = 32 fibers for CFA; data obtained from three animals in each group). The lack of a strong sensitization in aged animals following an acute inflammatory injury may reflect the fact that systemic inflammation increases with age (Singh and Newman, 2011): aged animals may already have an elevated level of inflammation compared with young animals, such that an additional inflammatory load has limited effects. This hypothesis is supported by recordings of C fibers from uninjured young and aged mice, as action potential firing in response to a mechanical stimulus was significantly higher in uninjured aged animals compared with uninjured young animals (Fig. 2D ^m^; ^*^p < 0.05 with two-way ANOVA; ^###^*p* < 0.001 with Bonferroni *post hoc* test; *n* = 25 fibers for both aged and young, three animals in each group). The age-dependent differences in baseline afferent firing also mirror our behavioral observations (Fig. 1A), whereby aged control mice exhibited greater mechanical sensitivity at baseline compared with young control mice.

We also examined von Frey thresholds of isolated C fibers from acutely inflamed and control animals. Despite marked reductions in behavioral von Frey thresholds in both age groups after 2 days of CFA inflammation, von Frey thresholds of individual C fibers in the skin–nerve preparation were unchanged in either cohort following acute inflammation (Fig. 2E ^n^; *p* > 0.05 with Kruskal–Wallis test; *n* = 25 fibers for young PBS group, *n* = 28 fibers for young CFA group, *n* = 25 fibers for aged PBS group, and *n* = 32 fibers for aged CFA group; three animals in each group). In fact, von Frey thresholds of individual C fibers were similar between PBS-injected aged and young mice, despite the differences in mechanical paw withdrawal thresholds between these cohorts at baseline (Fig. 1A). These seemingly disparate findings in von Frey threshold measures between single afferent fibers and behavioral responses may reflect the fact that mechanical stimulation on the behavioral level activates many different fiber types with overlapping receptive fields whose responses are all integrated at the spinal and brain levels, while skin–nerve preparations entail recordings from the receptive field of only one fiber at a time. Alternatively, these findings may also be the result of testing the glabrous skin behaviorally and recording from afferents innervating the hairy skin in the *ex vivo* skin–nerve preparation.

Additionally, we also examined the ongoing discharge of C fibers from acutely inflamed animals, as this type of activity may partially mediate non-evoked pain (Bennett, 2012). Ongoing discharge was classified as a firing rate >0.05 Hz (six action potentials over a 2 min interval). We found that a higher percentage of C fibers from inflamed preparations exhibited spontaneous activity in both young and aged animals (∼60% of fibers in CFA-inflamed preparations and ∼30% in PBS-injected control preparations), although this relationship was not statistically significant (Fig. 2F ^o^; *p* > 0.05 with Fisher’s exact test, for both young and aged animals; *n* = 25-32 fibers, as noted previously; three animals in each group). Conduction velocities were slightly different for C fibers from young, PBS-injected animals (0.46 ± 0.03 m/s) compared with C fibers from young, CFA-injected animals (0.62 ± 0.04 m/s; ^**^p < 0.01, Student’s *t* test), but no differences were noted in the conduction velocities of aged C fibers from the CFA- and PBS-treated groups or when comparing the aged PBS group to the young PBS group (data not shown).

### Young, but not aged, C-fiber nociceptors are inhibited during chronic inflammation

**Figure 3.**
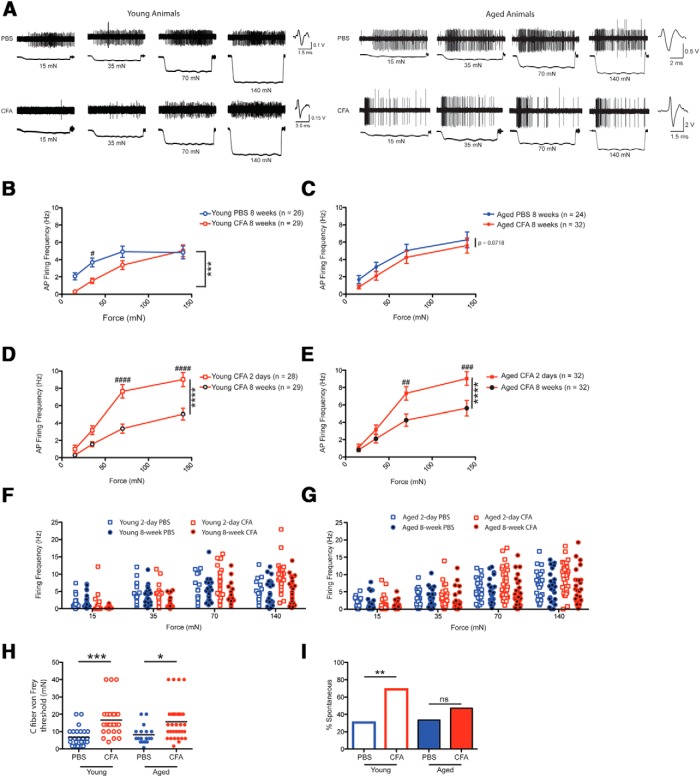
Chronic inflammation results in a desensitization of C fibers to mechanical force in young, but not aged animals. ***A***, Trace examples from young animals injected with either PBS (top left) or CFA (bottom left) and aged animals injected with either PBS (top right) or CFA (bottom right). ***B***, After 8 weeks of inflammation, C fibers from young animals respond with significantly lower action potential firing rates in response to increasing mechanical forces. ***C***, After 8 weeks of inflammation, C fibers from aged animals trend toward lower firing rates in response to increasing mechanical forces. ***D***, The firing rates of C fibers from inflamed young animals are significantly lower after 8 weeks of chronic inflammation compared with 2 days of acute inflammation. ***E***, The firing rates of C fibers from inflamed aged animals are significantly lower after 8 weeks of chronic inflammation compared with 2 days of acute inflammation. ***F***, ***G***, Plots of the firing rates of individual C fibers at different forces for each cohort for young (***F***) and aged (***G***) animals. Note that after 2 days of acute inflammation the entire population of C fibers in both young and aged animals shifts toward elevated firing rates, rather than only a subpopulation of increased responders. ***H***, von Frey thresholds for individual C fibers are elevated in both young and aged animals after 8 weeks of chronic inflammation. Each point on the graph represents the von Frey threshold of an individual C fiber, and the black bars are indicative of the group mean. ***I***, Chronic inflammation results in an increased percentage of C fibers demonstrating ongoing, nonevoked activity in young animals, but not in aged animals.

Although skin–nerve recordings from acutely inflamed animals showed intriguing differences between young and aged animals, we were particularly interested in the responses of C-fiber nociceptors during bona fide chronic pain, as this is a more pressing issue clinically than acute pain. Therefore, we also performed recordings from young and aged animals 8 weeks after CFA or PBS injection (Fig. 3A).

Strikingly, C fibers from CFA-injected animals actually exhibited a reduction in firing rates compared with PBS-injected controls at the 8 week time point in young animals, with the reduction in firing most evident at the lowest force values (Fig. 3B ^p^; ^***^p < 0.001 with two-way ANOVA; ^#^*p* < 0.05 with Bonferroni *post hoc* analysis; *n* = 26 and 29 fibers; *n* = 4 animals for PBS group and *n* = 6 animals for CFA group). In aged animals, chronic CFA-mediated inflammation had no effect on C fiber firing in comparison with PBS-injected controls (Fig. 3C ^q^; *p* > 0.05 with two-way ANOVA; *n* = 24 and 32 fibers; 10 animals for PBS group and 7 animals for CFA group). Importantly, for both young and aged animals, firing from chronically inflamed C fibers was significantly lower than the firing from acutely inflamed C fibers throughout the force series (Fig. 3D ^r^,*E* ^s^; ^****^p < 0.0001 with two-way ANOVA; ^##^*p* < 0.01 and ^####^*p* < 0.0001 with Bonferroni *post hoc* test; *n* = 28-32 fibers, as noted in Fig. 3D ^r^,*E* ^s^). These findings were incredibly surprising because chronically inflamed young and aged animals displayed continued, prominent behavioral sensitization to mechanical stimuli at this 8 week chronic time point (Fig. 1). Interestingly, when we examined the firing rates of individual C fibers at each force, we noted that acute, 2 d CFA-mediated inflammation results in a population-wide shift toward elevated firing rates in both young and aged animals (Fig. 3F,*G*). Recent research (Lennertz et al., 2012) has indicated that C-fiber sensitization following inflammation is mediated entirely by a population of C fibers that is responsive to both cold and mechanical, but not heat, stimulation. We did not test multiple modalities on individual C fibers in this study, but our finding that the entire population of C fibers responds with increased mechanical firing following acute inflammation argues that other populations of C fibers, including the C-mechano-only, C-mechano-heat-cold, and C-mechano-heat subtypes, are also likely to be sensitized to mechanical force following inflammation.

Additionally, we examined von Frey thresholds of isolated C fibers from young and aged animals after 8 weeks of CFA-mediated inflammation. Although we found no differences in von Frey thresholds between CFA-injected animals and PBS-injected controls at the 2 day time point (Fig. 2E), at 8 weeks we unexpectedly found significant elevations in von Frey thresholds of C fibers obtained from both young and aged inflamed mice (Fig. 3H ^t^; *p* < 0.0001 overall with Kruskal–Wallis test; ^***^p < 0.001, ^*^p < 0.05 with Bonferroni *post hoc* analysis; *n* = 26-32 fibers, as previously indicated; 4-10 animals, as previously indicated). Together, the elevated von Frey thresholds and reduced suprathreshold firing of C fibers after 8 weeks of inflammation in young animals suggest that a previously unreported plasticity is occurring in nociceptors of chronically inflamed young animals. In a similar vein, the elevated von Frey thresholds and trend toward reduced suprathreshold firing for aged C fibers points toward a similar, albeit weakened, phenomenon in aged animals.

Interestingly, despite the apparent reduction in action potential firing in response to evoked mechanical stimuli at 8 weeks of chronic inflammation, we did observe a significant elevation in the number of C-fiber afferents displaying spontaneous firing in young animals at this time point (Fig. 3I ^u^; ^**^p < 0.01 for young and *p* > 0.05 for aged with Fisher’s exact test; *n* = 26-32 fibers, as previously indicated; 4-10 animals, as previously indicated). Thus, at least in young animals, spontaneous chronic pain may still be mediated by ongoing discharge of peripheral afferents. Additionally, no differences in conduction velocity were noted between any of the cohorts.

### Firing patterns in C fibers are unchanged during chronic inflammation

Given the continued behavioral sensitization to mechanical stimuli, it was surprising that nociceptor firing would be so strongly reduced in both young and aged animals 8 weeks after CFA injection compared with 2 days post-CFA injection (Fig. 3D,*E*). In our view, the following three leading possibilities could explain this phenomenon: (1) the pain behaviors displayed by chronically inflamed mice were solely dependent on plasticity in the CNS (central sensitization); (2) painful information during chronic inflammation is propagated to the CNS along a different type of peripheral afferent; or (3) peripheral afferent communication of painful information to the spinal cord depends on a mechanism other than the absolute number of action potentials propagated, such as firing patterns or spike timing.

**Figure 4.**
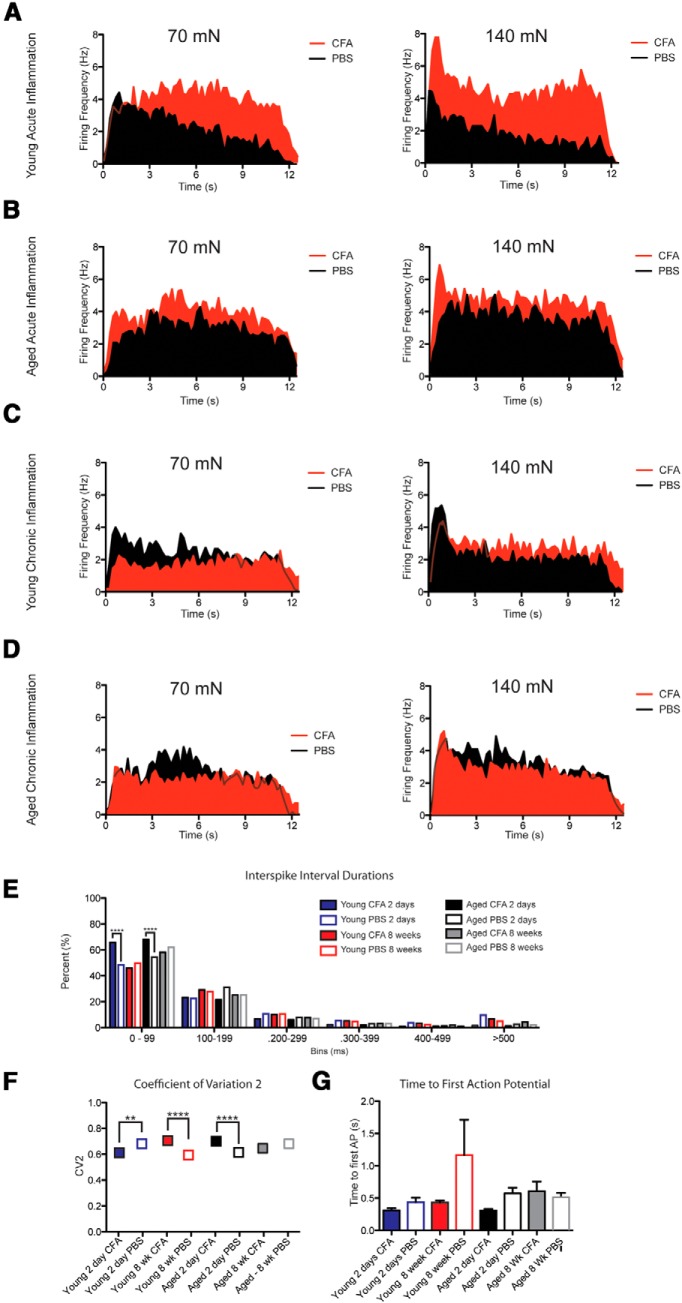
C-fiber action potential firing patterns do not explain the significant behavioral sensitization, but reduction in action potential firing rates during chronic inflammation. ***A–D***, Grouped instantaneous firing rates over the 12 s mechanical stimulus binned into 200 ms intervals for fibers from young acutely inflamed animals (***A***), aged acutely inflamed animals (***B***), young chronically inflamed animals (***C***), and aged chronically inflamed animals (***D***). ***E***, C fibers from acutely inflamed young and aged animals fired with a significantly higher percentage of interspike intervals between 0 and 99 ms. ***F***, The CV_2_ for a 140 mN stimulus were significantly different for C fibers from acutely inflamed young and aged animals, and chronically inflamed young animals, but these relationships do not consistently demonstrate that variability may underlie the increased behavioral sensitization seen acutely and chronically. ***G***, The time to first action potential after the onset of the mechanical stimulus is not different for any of the cohorts.

How different signals are communicated to the CNS has not yet been fully resolved, but some studies (Wan et al., 2000; Tanner et al., 2003) have indicated that the spike timing of action potentials is an important component of pain sensation. To explore the possibility that sensations of pain are communicated to the CNS via spike patterns during situations of chronic pain, rather than just the overall firing rate, we first examined the plots of instantaneous firing frequency over time (Fig. 4A*–D*). For young animals during acute inflammation, we observed elevated instantaneous firing rates compared with PBS controls throughout the 12 s duration of the mechanical stimulus; additionally, there appeared to be a lack of adaptation by C fibers from acutely inflamed preparations compared with controls (Fig. 4A). C fibers from acutely inflamed aged animals behaved similarly in that firing was elevated throughout the stimulus, but fibers from inflamed and control preparations seemed to adapt equivalently (Fig. 4B). When examining chronic time points for C fibers from young and aged animals, we saw much of the same phenomenon: although C fibers fired fewer action potentials than during acute inflammation, these recordings showed adaptation and firing throughout the stimulus that were similar to those of PBS controls for both age groups (Fig. 4C,*D*).

Since there were no consistent differences in firing adaptation during the mechanical stimulus, we next decided to examine whether fibers exposed to chronic inflammation fired with shorter ISIs. Some studies examining action potential firing in a variety of pain models found that subsets of C fibers fired more action potentials with short, 100-200 ms intervals between successive spikes (Chen and Levine, 2003, 2007; Tanner et al., 2003). The specific timing of action potentials within a train has also been shown to be important in systems such as the whisker barrel column of the somatosensory cortex in rats (Panzeri et al., 2001). When we examined the responses of C fibers to a 140 mN stimulus, we found that while acute inflammation resulted in a significantly higher percentage of ISIs in the 0-99 ms range (65.6% vs 45.9% for young and 68.06% vs 59.7% for aged), there was no difference between the CFA and PBS groups 8 weeks after injection in either young or aged mice (Fig. 4E ^v^; ^****^p < 0.0001, χ^2^ test with subsequent Fisher’s exact test for individual comparisons; *n* = 1082–2971 total ISIs per group). Thus, chronic inflammatory pain is unlikely to be communicated based on the rapidity with which C-fiber nociceptors fire within a given spike train.

We next reasoned that a message of pain could conceivably be communicated to the CNS by the timing or variability in the timing of action potentials within the spike train. Indeed, some researchers have postulated that the brain actually uses variability in action potential timing to alter the probability that neurotransmitters are released at a given synapse (Smetters and Zador, 1996). Models from computational studies have shown that seemingly variable action potential firing patterns may contain important contextual information that other neurons are able to decode (Softky, 1995). Furthermore, the CNS may differentiate input from different end organs in the skin based on the variability of firing within their action potential trains (Wellnitz et al., 2010). Therefore, we measured the CV_2_ (see Materials and Methods; Chen and Levine, 2003, 2007; Tanner et al., 2003) for every interspike interval within a given cohort, with higher values indicating more variability in the spike timing for a given action potential train (Fig. 4F ^w^; ^****^p < 0.0001 with one-way ANOVA; ^##^*p* < 0.01 and ^####^*p* < 0.0001 with Bonferroni *post hoc* test; *n* = 808–2001 ISIs). Although we found differences between fibers from CFA-inflamed preparations and their controls for three of the four cohorts in response to a 140 mN stimulus, the changes we observed were not consistent. For instance, C fibers from inflamed young animals at the acute time point exhibited less variability (0.61) than their PBS controls (0.68), while the opposite was true for C fibers from inflamed aged animals at the acute time point (0.70 vs 0.61 for aged PBS controls). However, by 8 weeks C fibers from chronically inflamed young animals exhibited more variability than their PBS controls, and no difference was found between the PBS and CFA groups for aged animals. Thus, the variability in action potential firing, which could conceivably code messages of pain due to mild oscillating or bursting behavior, also cannot explain how chronically inflamed animals are able to exhibit pain behaviors despite the markedly reduced action potential firing rates in primary afferent fibers.

Finally, we decided to examine the time from onset of our mechanical stimulus to firing of the first action potential in the train, since other somatosensory research has found that the time from mechanical stimulus onset to first spike generation by low-threshold mechanoreceptive afferents is critical for encoding tactile information (Johansson and Birznieks, 2004). Again, we found no difference between specific groups for this measure (Fig. 4G ^x^; *p* < 0.05 overall with one-way ANOVA; *n* = 23-31 fibers, no specific differences with Bonferroni *post hoc*), making it unlikely that pain is simply coded by the timing of the first action potential in response to a stimulus.

Collectively, these data, coupled with our recordings from primary afferents showing reduced firing during chronic inflammation, suggest that alterations in C-fiber activity patterns or timing of impulses do not contribute to pain sensation during a chronic inflammatory state in either young or aged animals.

### C-fiber responses to chemical agonists are also reduced after 8 weeks of chronic inflammation

Because our data strongly suggested that chronic inflammation causes reduced afferent drive to the CNS in response to mechanical stimuli in young animals, we next asked whether this phenomenon could be generalized to other types of somatosensory stimuli. Therefore, we decided to test the responsiveness of C fibers to the potent transient receptor potential vanilloid 1 (TRPV1) agonist capsaicin. TRPV1 is located on 33-45% of small-diameter neurons (Breese et al., 2005; Cavanaugh et al., 2011), and capsaicin generates a robust calcium influx and action potential trains when applied to the cell body or afferent terminals, respectively (Caterina et al., 1997; Seabrook et al., 2002; Carlton et al., 2004; Correll et al., 2004; Barabas and Stucky, 2013). Importantly, in an effort to record from the same population of C fibers, these experiments used only C fibers that were responsive to mechanical stimuli and excluded mechanically insensitive fibers.

**Figure 5.**
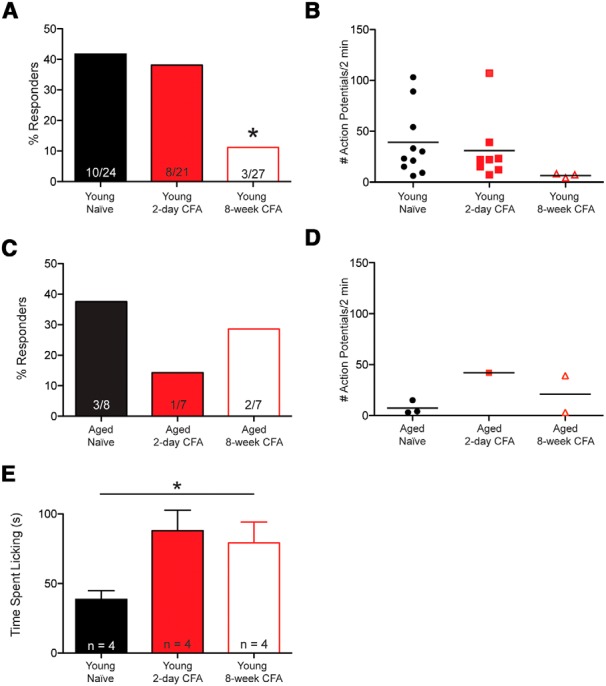
C-fiber responses to capsaicin are reduced during chronic inflammation, while behavioral sensitization to capsaicin remains intact. ***A***, C-fiber responses to capsaicin are similar under naïve and acutely inflamed conditions in young animals, but responses are strongly attenuated during chronic inflammation. ***B***, The number of action potentials fired by capsaicin-sensitive C fibers is also reduced after 8 weeks of chronic inflammation in young animals (although this is not statistically significant). ***C***, In aged animals, C-fiber responses to capsaicin are similar across the naïve, acute inflamed, and chronic inflamed states. Note the low numbers that are due to the lack of aged animal availability. ***D***, Number of action potentials fired by aged C fibers in response to capsaicin. ***E***, Young animals exhibit sensitized pain behaviors in response to capsaicin injection during both acute inflammatory and chronic inflammatory states, despite the reduced afferent responses to capsaicin at 8 weeks.

In young naïve animals, we found that 41.7% of C fibers fired at least three action potentials in response to incubation with 10 μm capsaicin for 2 min, with an average of 38.3 ± 10.6 action potentials generated (Fig. 5A,*B*; *n* = 10 of 24 fibers; data from four animals). After 2 days of acute inflammation, we found that a similar percentage of C fibers from young animals responded to capsaicin with comparable firing rates (38.09% responders, 30.88 ± 14.4 action potentials, Fig. 5A,*B*; *n* = 8 of 21 fibers, data from three animals). Although we could not find any other studies that had tested the responsiveness of C fibers to capsaicin in the skin–nerve preparation after CFA-mediated inflammation, the lack of sensitization (either in the percentage of responders or the magnitude of the firing rate) was surprising in light of studies demonstrating sensitization of the cell body to capsaicin after acute inflammation (Breese et al., 2005; De Souza et al., 2013). However, after 8 weeks of chronic inflammation, we observed a strong reduction in responses to capsaicin that was reminiscent of the reduced mechanically-induced firing observed at this time point (11.1% of responders, 6.33 ± 1.20 action potentials, Fig. 5A ^y^,*B* ^z^; 3 of 27 fibers, data from four animals; *p* < 0.05 overall with χ^2^ test; ^*^p < 0.05 for naïve vs 8 weeks of inflammation, and for 2 days vs 8 weeks of inflammation with Fisher’s exact test). Additionally, no differences in conduction velocity were noted between any cohort.

Similar teased fiber experiments were also performed in aged animals. At baseline, 37.5% of C fibers from aged animals responded to capsaicin incubation with action potential firing (Fig. 5C ^aa^; three of eight fibers, data from two animals). After 2 days of acute inflammation, 14.3% of fibers responded, while after 8 weeks of chronic inflammation, 28.6% of C fibers responded to capsaicin (Fig. 5C; one of seven fibers for the acute group and 2 of seven fibers for the chronic group; data from two animals). We advise caution in interpreting these findings from aged animals, as they are derived from low numbers (seven to eight fibers per group) due to limitations in the availability of animals >18 months of age in our animal colony. However, it is interesting to note the number of C fibers that were responsive to capsaicin after 8 weeks of chronic inflammation in aged animals (2 of 7 fibers) compared with C fibers from young animals at the same time point (3 of 27 fibers). Grossly, the percentage of responders to capsaicin reflects the responsiveness of C fibers to mechanical stimulation at the chronic time point: in young animals, there is a generalized reduction in responsiveness, while in aged animals there is only a slight, nonsignificant reduction in responsiveness to somatosensory stimuli. Additionally, no differences in conduction velocity were noted between any of the cohorts.

Importantly, we also tested the behavioral responses to capsaicin for another cohort of animals at the naïve, acute inflammatory, and chronic inflammatory time points. As expected, young animals experiencing both acute 2 day inflammation and chronic 8 week inflammation exhibited sensitized responses to 100 μm capsaicin injection compared with naïve animals (Fig. 5E ^bb^; ^*^p < 0.05 overall with one-way ANOVA; four animals per group). This corresponds well with our mechanical data at the behavioral and afferent levels, as chronically inflamed animals continued to show strongly sensitized pain behaviors despite reduced afferent responsiveness. Thus, we conclude that chronic inflammation mediates a global reduction in afferent drive in nociceptive C fibers that is not modality specific.

### AM fibers also exhibit reduced drive after 8 weeks of chronic inflammation

Our data convincingly provides evidence that C fibers are desensitized to multiple modalities as a result of chronic inflammation, in spite of continued behavioral sensitization to these modalities. Although C fibers have been the most studied class of afferents with regard to pain, we wondered whether chronic pain could be mediated by Aδ nociceptors, since this population of afferents also transmits sensations of mechanical pain. Therefore, we decided to examine the responsiveness of Aδ nociceptors (AMs) to mechanical stimuli under naïve, acute inflammatory, and chronic inflammatory conditions in young animals (these experiments could not be performed in aged animals due to a lack of aged animals in our colony).

**Figure 6.**
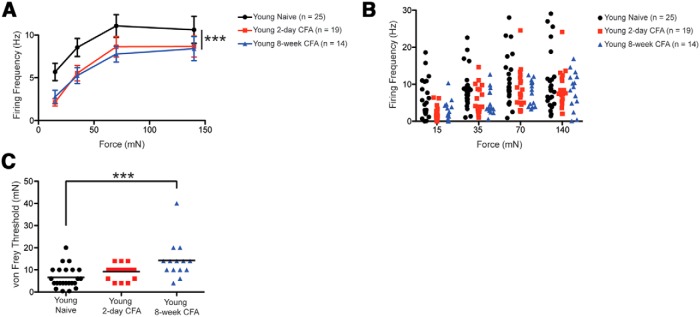
AM fibers from young animals exhibit reduced mechanical firing rates following inflammation. ***A***, Following both 2 day acute and 8 week chronic inflammation, AM fibers from young animals exhibit reduced firing rates in response to mechanical stimuli. ***B***, Plots of the firing rates of individual AM fibers at different forces for each cohort of young animals. Note the loss of a population of high-responding AM fibers at the 2 day and 8 week time points. ***C***, von Frey thresholds of individual AM fibers from young animals are elevated after 8 weeks of chronic inflammation compared with fibers from naïve animals.

Similar to chronically inflamed C fibers, we found that chronically inflamed AM fibers from young animals also exhibited a significant reduction in firing rates in response to a series of increasing mechanical forces (Fig. 6A ^cc^; ^***^p < 0.001, two-way ANOVA overall; *n* = 14-25 fibers, as indicated on graph; five animals for the naïve and 2 day inflammation groups; four animals for the 8 week inflammation group). Surprisingly, we also observed a reduction in the firing of AM fibers after a 2 day acute inflammatory injury (Fig. 6A). Other studies have shown either a sensitization of A fibers (Andrew and Greenspan, 1999; Potenzieri et al., 2008; Moshourab and Stein, 2012) or no change in the firing rates of Aδ fibers (Lennertz et al., 2012) after acute CFA-mediated inflammation. Interestingly, the results obtained from many of those studies examined A fibers in the glabrous skin of the hindpaw (Andrew and Greenspan, 1999; Potenzieri et al., 2008; Lennertz et al., 2012), while this study used inflamed hairy skin innervated by the saphenous nerve. We therefore cannot rule out the possibility that the responsiveness of A fibers is dependent on the type of skin (hairy or glabrous) that is innervated; indeed, a recent report (Weyer et al., 2015) has demonstrated that the target of innervation is critical for the mechanical responses of myelinated neurons to inflammatory stimuli. However, another report examining AM fibers from the rat hairy skin after acute (3-4 day) CFA-mediated inflammation also found sensitization to mechanical stimuli (Moshourab and Stein, 2012). Future AM recordings following inflammation must be performed to sort out this discrepancy.

When we plotted the responses of individual AM fibers to increasing force for each group, we noted that the difference between cohorts was really due to a selective loss of a population of AM fibers from the inflamed groups with extremely high response rates to mechanical stimuli that are present in the naïve group (Fig. 6B). At this point, our results cannot determine whether this subpopulation of AM fibers is rendered silent by inflammation, or whether the inflammatory process simply reduces the firing of this population to a level similar to other moderately firing AM fibers. However, this finding is striking when compared to C fibers, which displayed a population-wide shift toward higher firing frequencies following inflammation (Fig. 3F).

Also in accord with the findings from C fibers, AM fibers exhibited no change in von Frey thresholds after an acute inflammatory injury, but displayed significantly elevated von Frey thresholds after 8 weeks of chronic inflammation (Fig. 6C ^dd^; ^***^p < 0.001 with Kruskal–Wallis test; *n* = 14-25 fibers; five animals for the naïve and 2 day inflammation groups; four animals for the 8 week inflammation group). Additionally, no differences in conduction velocity were noted between any of the cohorts.

Collectively, these data demonstrate two important points. First, our data suggest that the behavioral hyperalgesia observed in response to mechanical stimulation during acute inflammation is dependent primarily on C fibers, and not on Aδ fibers, in the peripheral nervous system. Second, the continued behavioral sensitization during chronic inflammation is not dependent on elevated nociceptive afferent drive to the CNS, as both C fibers and AM fibers display elevations in their thresholds and reductions in suprathreshold firing rates at chronic time points.

### Changes in gene expression do not explain the reduced afferent firing during chronic inflammation

We next wondered what mechanisms underlie the changes in action potential firing at 2 days and 8 weeks post-CFA injection. We reasoned that changes in the gene expression of key mechanosensitive and voltage-gated ion channels in sensory neurons could cause the amplification of afferent firing we observed at 2 days and the reduction in firing at 8 weeks. Therefore, we began by examining the effects of acute and chronic inflammation on the expression of voltage-gated sodium channels specific to nociceptors (Cummins et al., 2007) in the left L2–L5 DRGs, which innervate the left hindpaw.

**Figure 7.**
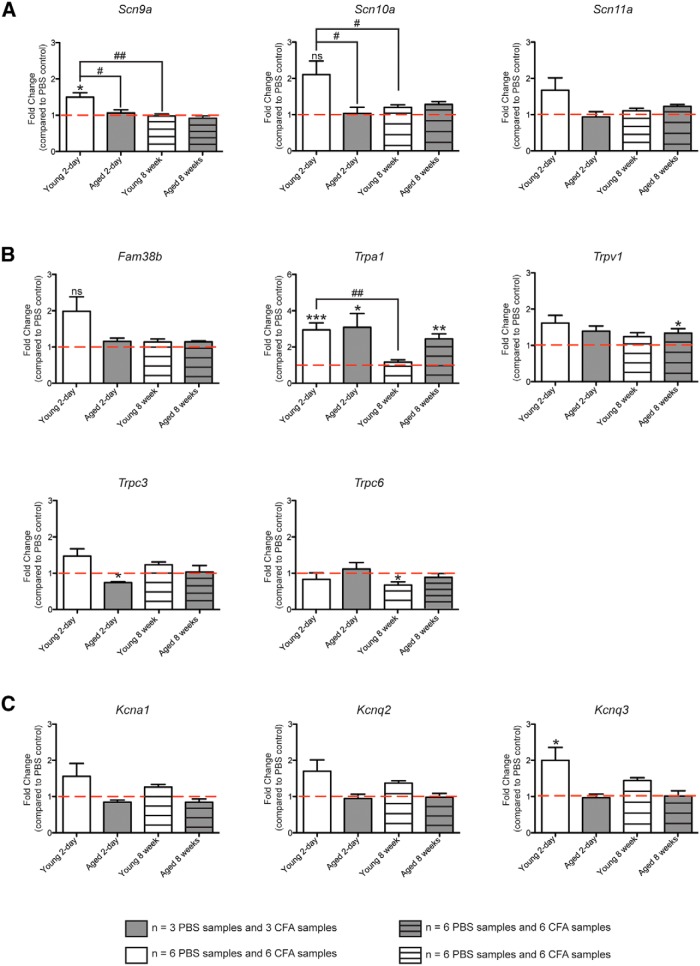
Changes in gene expression of voltage-gated and mechanosensitive ion channels do not explain the reduced action potential firing after 8 weeks of chronic inflammation. ***A***, Gene expression for voltage-gated sodium channels Na_V_1.7 (*Scn9a*), Na_V_1.8 (*Scn10a*), and Na_V_1.9 (*Scn11a*). Bars indicate the fold change of the CFA condition over the PBS condition for each cohort. The red dotted line indicates a fold change of 1, meaning no change in expression levels between CFA and PBS conditions. ^*^Indicates significant fold changes for the CFA vs PBS condition; ^#^indicates significant differences in the fold change between cohorts. ***B***, Gene expression (shown as fold change compared with PBS controls) for Piezo2 (*Fam38b*) and TRP channels. ***C***, Gene expression (shown as the fold change compared with PBS controls) for voltage-gated potassium channels K_V_1.1 (*Kcna1*), K_V_7.2 (*Kcnq2*), and K_V_7.3 (*Kcnq3*).

Previous research has demonstrated significant dysregulation of voltage-gated sodium channels in sensory neurons in a variety of pain models (Waxman et al., 2000; Craner et al., 2002). When compared with the cognate L2–L5 DRGs from PBS-injected controls (Fig. 7 ^ee,ff^, red lines), we found that *Scn9a* (Na_V_1.7) transcripts were significantly elevated by 1.5-fold in young mice 2 days after CFA injection, but found no differences in *Scn9a* expression in the DRGs of young mice after 8 weeks of inflammation or aged mice after 2 days or 8 weeks of inflammation compared with controls (Fig. 7A, left; ^*^p < 0.05 with Student’s *t* test, CFA vs PBS samples^ee^; *p* < 0.001 with one-way ANOVA for fold changes between groups^ff^; ^#^*p* < 0.05 and ^##^*p* < 0.01 with Bonferroni *post hoc* test; *n* = 3 animals for aging groups; *n* = 6 animals for young groups). We saw a similar trend for *Scn10a* (Na_V_1.8), with elevated expression of these transcripts compared with controls during acute inflammation in young mice, although these changes were not statistically significant due to increased variability (Fig. 7A, middle and right). Furthermore, we again found no differences in the expression of these channels in aged animals or in young animals after 8 weeks of inflammation.

Interestingly, the expression of all three voltage-gated sodium channels was unchanged in aged animals following acute inflammation compared with PBS-injected controls, which perhaps contributes to the lack of strong afferent sensitization to mechanical stimuli observed with teased fiber recordings at the 2 day time point in aged mice (Fig. 2C). Perhaps most importantly, however, was that the expression of *Scn9a*, *Scn10a*, and *Scn11a* was not different in chronically inflamed young and aged animals compared with PBS-injected controls at the 8 week time point. This suggests that the reduced action potential firing at chronic time points is not due to a decrease in the expression of these voltage-gated sodium channels.

Interestingly, although changes in voltage-gated sodium channels do not seem to underlie the reduced firing we observed in young animals after 8 weeks of chronic inflammation, the elevated expression we observed in these channels after 2 days of inflammation may explain why C fibers from this cohort exhibited elevated conduction velocities. Likewise, the lack of change in Na_V_ channel gene expression in aged animals after 2 days mirrors the lack of change in conduction velocity when recording from aged, acutely inflamed C fibers.

We also examined channels that have been linked to mechanotransduction, as alterations in the channels that sense the initial mechanical stimulus could have a large impact on the number of action potentials propagated in response to a given mechanical stimulus (Fig. 7B). Piezo2 (*Fam38b*), which is the major mechanotransducer in myelinated low-threshold mechanoreceptors (Ranade et al., 2014), had unaltered gene expression in the four cohorts (Fig. 7B ^ee,ff^, top left; *p* > 0.05 with Student’s *t* test for CFA vs PBS for each time point; *n* = 3 animals for aged groups and 6 animals for young groups). In contrast, transient receptor potential ankyrin 1 (TRPA1), which has been shown to be integral to the mechanical sensitization observed after an acute inflammatory insult (Lennertz et al., 2012), was found to be elevated threefold in both young and aged DRGs 2 days after CFA injection (Fig. 7B ^ee,ff^, top middle; ^*^p < 0.05, ^**^p < 0.01, ^***^p < 0.001; *n* = 3 aged animals; *n* = 6 young animals). Interestingly, *Trpa1* transcript levels remained elevated during chronic inflammation in aged animals, but not in young animals (*p* < 0.01 with one-way ANOVA for *Trpa1* expression levels; ^##^*p* < 0.01 with Bonferroni *post hoc* test). This mirrors recent behavioral findings indicating that TRPA1 is critical for chronic pain in aged animals, but only for acute pain in young animals (Garrison and Stucky, 2014).

TRPV1, which has widely been shown to be involved in pain sensation and may be activated by mechanical stimuli under some circumstances (Hillery et al., 2011; Julius, 2013), showed a small (33%), but significant, elevation 8 weeks after CFA injection in aged animals (Fig. 7B ^ee,ff^, top right; ^*^p < 0.05, Student’s *t* test; *n* = 3). Our data showed no change in *Trpv1* gene expression in young animals after 8 weeks of inflammation compared with controls, suggesting that the reduced afferent responsiveness to capsaicin (Fig. 5A,*B*) is not due to a reduction in *Trpv1* transcript expression. Transient receptor potential canonical (TRPC) 3, along with its family member TRPC6, has been linked to normal mechanotransduction in subsets of small-diameter neurons (Quick et al., 2012). *Trpc3* was reduced 28% in aged mice during acute inflammation, but levels were normalized by 8 weeks of inflammation (Fig. 7B ^ee,ff^, bottom left; ^*^p < 0.05 with Student’s *t* test, *n* = 3). *Trpc6* levels were reduced by one-third in young mice after 8 weeks of chronic inflammation (Fig. 7B ^ee,ff^, bottom middle; ^*^p < 0.05 with Student’s *t* test; *n* = 6).

Although some changes were noted in channels linked to mechanotransduction, none of the changes pointed to a clear explanation for the reduced firing observed after 8 weeks of chronic inflammation. We therefore examined whether potassium channels, which help to control the firing rates of nociceptors and may be dysregulated during painful injuries (Tsantoulas and McMahon, 2014), could have altered expression to account for the observed physiology. Transcript levels for *Kcna1* (K_V_1.1), which has recently been found to serve as a “brake” for mechanically gated currents in nociceptors (Hao et al., 2013), were unaltered in any of the four cohorts (Fig. 7C ^ee,ff^, left). We also chose to examine the expression of *Kcnq2* (K_V_7.2) and *Kcnq3* (K_V_7.3), which together mediate the “M” current in sensory neurons that constitutes the major subthreshold K^+^ current and may limit inflammatory pain when activated (Passmore et al., 2003). *Kcnq2* transcript levels were unaltered in any group, and *Kcnq3* transcript levels were elevated twofold only in young animals after 2 days of acute inflammation (Fig. 7C ^ee,ff^, middle and left; ^*^p < 0.05 with Student’s *t* test; *n* = 6 animals).

Cumulatively, these results argue against the hypothesis that alterations in *Scn9a*, *Scn10a*, *Scn11a*, *Trpa1*, *Fam38b*, *Trpv1*, *Trpc3*, *Trpc6*, *Kcna1*, *Kcnq2*, or *Kcnq3* gene expression underlie the reduced peripheral drive observed after 8 weeks of chronic inflammation compared with 2 days of acute inflammation in both young and aged animals.

## Discussion

These data highlight the novel finding that C-fiber nociceptors in young animals exhibit enhanced mechanical firing following an acute inflammatory injury, but reduced firing during the chronic inflammatory phase. Importantly, the reduced nociceptor firing observed chronically in response to both mechanical and chemical stimulation occurs despite continued prominent behavioral sensitization, suggesting that increased peripheral drive is necessary for the installation, but not the maintenance, of central sensitization in young animals. Additionally, reduced firing in AM afferents after 8 weeks of chronic inflammation suggests that reduced afferent drive during chronic pain is not C fiber specific, but rather a global mechanism in nociceptive afferents. In contrast to data from young animals, our results also suggest that aged animals are less malleable in response to an inflammatory injury: they exhibit less behavioral sensitization, and their C fibers fire at rates similar to those of controls during both acute and chronic inflammation.

### Rationale for reduced afferent firing after chronic inflammation

These findings are no doubt surprising given the large body of evidence examining peripheral mechanisms of pain under the assumption that input from peripheral afferents mediates and/or maintains chronic pain states. However, this finding is not unprecedented, as nociceptive afferent firing in response to mechanical stimulation has been shown to be reduced following a chronic constriction injury (Schmidt et al., 2012).

We initially speculated that the desensitization of C fibers and AM fibers in response to mechanical stimuli as a result of chronic inflammation was due to changes in the gene expression of voltage-gated or mechanosensitive ion channels. However, none of the channels we examined displayed changes in gene expression that could account for the reduction in firing. This does not, of course, preclude the possibility that unexamined channels are responsible for the changes, or that protein levels or channel functionality are altered following chronic inflammation. Additionally, an alternative explanation is that low-threshold mechanoreceptors may be sensitized during chronic pain states and are responsible for the majority of the pain phenotype observed. Some evidence suggests that this occurs in nerve injury models, where myelinated afferents have been shown to be critically important for tactile allodynia and hyperalgesia (Campbell et al., 1988; Sun et al., 2001; King et al., 2011; Boada et al., 2014).

Altogether, our data illustrating reduced nociceptive afferent firing points to a novel plasticity in C and AM fibers that has not been previously documented in chronic inflammatory pain. Therefore, we propose that the reduced peripheral drive at this time point serves to limit the amount of painful afferent information carried to the CNS. It is well documented that central sensitization, a form of central plasticity at nociceptive synapses, is a crucial component of chronic pain (Woolf, 2011). Since this plasticity can result in an increased probability of synaptic vesicle release per action potential volley (Schulz, 1997), it follows that the body's attempt to limit pain transmission would occur via a reduction in the number of action potentials reaching the central synapse.

### Alterations in C-fiber firing during chronic pain depend on disease pathology

It is also interesting to contrast this work with primary afferent recordings performed in other models of chronic pain. In another model with a persistent inflammatory component, a mouse model of sickle cell disease, animals experience chronic pain throughout their lives as a result of frequent hypoxic events, but C-fiber recordings exhibit consistent mechanical sensitization compared with controls (Hillery et al., 2011). In studies of neuropathic pain using the spared nerve injury model or spinal nerve ligation model, recordings of C fibers at chronic time points demonstrated significant sensitization to mechanical stimuli (Shim et al., 2005; Smith et al., 2013). In contrast, another study examining the mechanical sensitivity of C fibers following a chronic constriction injury found that afferent firing was reduced in response to mechanical stimuli (Schmidt et al., 2012). Thus, the response of C fibers to pain critically relies on both the time since injury induction and the etiology of the injury.

It is important to note that a recent study from our lab (Garrison and Stucky, 2014) examined the role of TRPA1 in chronic inflammatory pain in an aging model. Interestingly, that study found that C fibers from both young and aged mice exhibited *sensitization* to mechanical stimuli at 8 weeks after CFA inflammation, and that this sensitization was dependent on TRPA1 in aged animals. These findings are contrary to those presented in the current study, where we have found reduced firing of nociceptors at chronic time points in young animals and minimal changes in afferents from aged animals at acute or chronic time points. It is difficult to discern exactly why the results differ between studies, but several key differences may contribute. The current study uses substantially greater numbers, which decrease the risk of a type I error. The current study also uses male mice exclusively, while Garrison and Stucky (2014) largely used recordings from female mice. Given the wide body of data showing that sex can affect afferent responses to pain, this is a crucial difference (Mogil, 2012; Bartley and Fillingim, 2013). Finally, it should be noted that the background strains of the mice used in each study were different; mice used in the study by Garrison and Stucky (2014) were C57BL/6 mice, while the majority of mice used in this study were on a mixed C57BL/6/Swiss Webster/CBA background.

### Gene expression of key ion channels is largely unchanged during chronic inflammation

Although we were unable to identify a specific gene responsible for the reduced action potential firing during chronic inflammation, it is interesting to make note of the overall trends observed among the different groups. Most of the examined genes were elevated at the 2 day time point in young animals, suggesting that young animals are able to quickly alter gene expression in sensory neurons in response to an injury. In stark contrast, aged animals displayed minimal changes in gene expression at this same time point. While a different set of genes may display altered expression in aged animals than those examined in this study, it is intriguing to speculate that acute pain sensation may occur via a different mechanism in aged animals than in young animals. Interestingly, the sole strongly induced gene during acute inflammation in aged animals was *Trpa1*, which has previously been shown to be important for both acute and chronic pain behaviors in aged animals (Garrison and Stucky, 2014).

At chronic time points, young mice showed a general shift back toward baseline for gene expression levels; in fact, the only notable difference was a slight reduction in the expression of *Trpc6* in chronically inflamed animals compared with PBS-injected controls. Gene expression was largely the same for aged animals between the CFA and PBS groups at chronic time points, with the exception of *Trpa1* and *Trpv1*. It is interesting that *Trpv1* was found to be expressed at higher levels only in aged animals based on a recent report (Schwartz et al., 2013) that both TRPA1 and TRPV1 are important for the transition from acute to chronic pancreatic pain in young animals. Studies using a global TRPA1 knock-out mouse line and specific TRPA1 antagonists have demonstrated that a removal/blockade of TRPA1 reduces nociceptive primary afferent firing (Brierley et al., 2009; Kerstein et al., 2009; Kwan et al., 2009). Therefore, it could be expected that elevations in the gene expression of *Trpa1* in aged animals at chronic time points would subsequently result in elevated C-fiber firing rates. However, it is also known that TRPA1 plays an important role at the central synapse between nociceptive primary afferents and neurons in lamina I/II of the dorsal horn (Pertovaara and Koivisto, 2011; Sisignano et al., 2012). This raises the possibility that the role of TRPA1 in chronic pain in aged animals is not at the afferent terminals in the skin, but rather at the central terminal to promote greater fidelity at nociceptive synapses.

### Correlation with clinical literature

The clinical literature paradoxically shows that while aged individuals have decreased tactile sensitivity (Thornbury and Mistretta, 1981), higher percentages of aged individuals have complaints of pain (Krueger and Stone, 2008; Maxwell et al., 2008). Furthermore, aged individuals have reduced mechanical pain thresholds experimentally (Lautenbacher et al., 2005). Our data in aged mice show the opposite with regard to tactile sensitivity: aged mice have increased sensitivity at baseline based on von Frey thresholds. Additionally, while aged mice in this study exhibited significant pain behaviors following CFA inflammation, they actually exhibited reduced allodynia compared with young animals injected with CFA, as judged by paw withdrawal thresholds. However, hyperalgesia, as measured by responses to a suprathreshold stimulus, were similar at chronic time points for both young and aged animals. This mirrors what is observed clinically with aged individuals and young individuals who complain of pain reporting similar pain levels (Krueger and Stone, 2008).

In contrast to our findings that afferent drive is either unchanged or reduced compared with controls at chronic time points, clinical studies seem to validate the idea that peripheral afferent input must remain elevated during chronic pain. Evidence for this stems from examples such as the elimination of chronic pain in patients with osteoarthritis who undergo total knee arthroplasties or patients with chronic pain who experience relief following the application of topical lidocaine (Richards and McMahon, 2013). However, these studies do not discriminate between reducing enhanced activity of a sensitized nerve and reducing the normal activity of a nonsensitized nerve.

For instance, topical lidocaine has been shown to reduce pain in patients with peripheral neuropathic pain syndromes (Meier et al., 2003). Yet, lidocaine may reduce chronic pain in some patients, not because it returns elevated peripheral drive to baseline, but rather because it blocks all input from a peripheral neuron from ever reaching a central neuron. Indeed, applying a lidocaine patch to a healthy individual will also be efficacious because it blocks the transmission of sensory information. Likewise, a joint replacement may result in decreased pain because nerve fibers are no longer present in the joint to transmit any sort of sensory signal.

There has been some suggestion that age-related pain may be due to reduced descending inhibition in aged adults (Edwards et al., 2003; Riley et al., 2010; Marouf et al., 2014). While examining central mechanisms is outside the scope of the current study, our results suggest that at least some of the elevated acute pain in aged individuals may be the result of peripheral mechanisms. Nociceptive primary afferents exhibited a strong trend toward increased firing in aged animals following acute inflammatory injury, and changes in *Trpa1* gene levels were noted at this time point as well. However, given the overall blunting of the sensitization of primary afferents and the relative lack of changes in gene expression of nociceptive ion channels, it is possible that central mechanisms account for a large part of the acute pain response in this population.

### Conclusion

Collectively, the results of this study question whether it is pertinent to examine mechanisms of pain sensation in the peripheral nervous system using acute inflammatory models, since nociceptive C and AM fibers seem to contribute minimally, if at all, to chronic inflammatory pain. Indeed, this point is buoyed by recent research examining the role of leukocyte elastase in a model of neuropathic injury (Vicuña et al., 2015). That study demonstrated that inhibiting leukocyte elastase is effective at blocking pain acutely, but has no effect on pain sensation at chronic time points. Finding the molecular cause of the reduced action potential firing at chronic time points may, however, lead to new therapies if this process can be taken advantage of during the acute pain phase prior to the installation of chronic pain.

Our findings also shed light on the processes that may contribute to differences in pain sensation between young and aged populations, and should serve as the impetus for future mechanistic research into this understudied area.

**Table 2.** Statistical tests utilized in this manuscript

	**Data Structure**	**Type of Test**	**95% Confidence Interval**			
*a*	Non-normally distributed	Mann Whitney Test	−1.982 to 0			
*b*	Normally Distributed	2-way repeated measures ANOVA	BL		−1.786 to 0.9921	
			2 Days		1.399 to 4.177	
			2 Weeks		1.116 to 3.894	
			3 Week		0.6599 to 3.438	
			4 Weeks		1.513 to 4.291	
			6 Weeks		1.053 to 3.831	
			8 Weeks		0.9196 to 3.698	
*c*	Normally Distributed	2-way repeated measures ANOVA	BL		−1.895 to 1.047	
			2 Days		−3.114 to −0.1722	
			2 Weeks		−3.073 to −0.1315	
			3 Weeks		−3.467 to −0.5257	
			4 Weeks		−3.824 to −0.8828	
			6 Weeks		−3.708 to −0.7663	
			8 Weeks		−3.797 to −0.8067	
*d*	Normally Distributed	2-way repeated measures ANOVA	BL		−2.017 to −0.6001	
			2 Days		−0.1975 to 1.219	
			2 Weeks		0.2870 to 1.704	
			3 Weeks		−0.1994 to 1.218	
			4 Weeks		−0.1833 to 1.234	
			6 Weeks		−0.2278 to 1.189	
			8 Weeks		−0.5649 to 0.8520	
*e*	Normally Distributed	2-way repeated measures ANOVA	BL		−6.579 to 70.27	
			2 Days		15.50 to 92.35	
			2 Weeks		−2.743 to 74.11	
			3 Weeks		−0.06598 to 76.78	
			4 Weeks		−0.1534 to 76.70	
			6 Weeks		−18.63 to 58.22	
			8 Weeks		−6.579 to 70.27	
*f*	Normally Distributed	Student’s t-test	−12.04 to 8.812			
*g*	Normally Distributed	2-way repeated measures ANOVA	BL		−19.12 to 9.119	
			2 Days		31.71 to 59.95	
			2 Weeks		27.96 to 56.20	
			3 Weeks		35.05 to 63.29	
			4 Weeks		31.30 to 59.54	
			6 Weeks		31.30 to 59.54	
			8 Weeks		31.30 to 59.54	
*h*	Normally Distributed	2-way repeated measures ANOVA	BL		−30.63 to 19.91	
			2 Days		−20.39 to 30.15	
			2 Weeks		−14.44 to 36.10	
			3 Weeks		7.705 to 58.25	
			4 Weeks		15.32 to 65.87	
			6 Weeks		11.63 to 62.18	
			8 Weeks		10.93 to 62.31	
*i*	Normally Distributed	2-way repeated measures ANOVA	BL		−17.81 to 23.52	
			2 Days		1.122 to 42.45	
			2 Weeks		8.086 to 49.41	
			3 Weeks		−10.31 to 31.02	
			4 Weeks		−13.34 to 27.99	
			6 Weeks		−10.49 to 30.84	
			8 Weeks		−22.63 to 18.70	
*j*	Normally Distributed	1-way ANOVA	0.8595 to 1.648 for Young height			
			0.2179 to 1.028 for Aged height			
			0.3473 to 1.482 for Young width			
			0.4451 to 1.610 for Aged height			
*k*	Normally Distributed	2-way ANOVA	15.00 mN		−2.979 to 1.547	
			35.00 mN		−2.442 to 2.084	
			70.00 mN		1.031 to 5.556	
			140.0 mN		3.124 to 7.650	
*l*	Normally Distributed	2-way ANOVA	15.00 mN		−2.376 to 2.028	
			35.00 mN		−2.117 to 2.287	
			70.00 mN		−0.5504 to 3.854	
			140.0 mN		−0.3346 to 4.101	
*m*	Normally Distributed	2-way ANOVA	15.00 mN		−1.645 to 2.507	
			35.00 mN		−1.791 to 2.361	
			70.00 mN		−3.398 to 0.7536	
			140.0 mN		−5.603 to −1.451	
*n*	Non-Normally Distributed	Kruskal-Wallis Test	Aged CFA − 2 days vs Aged PBS − 2 days		−0.3564 to 0.4208	
			Aged CFA − 2 days vs Young CFA − 2 days		−0.3346 to 0.4188	
			Aged CFA − 2 days vs Young PBS − 2 days		−0.2396 to 0.5376	
			Aged PBS − 2 days vs Young CFA − 2 days		−0.3906 to 0.4105	
			Aged PBS − 2 days vs Young PBS − 2 days		−0.2949 to 0.5285	
			Young CFA − 2 days vs Young PBS − 2 days		−0.2937 to 0.5074	
*o*	Non-Normally Distributed	Contingency Table followed by Fisher’s Exact Test	1.081 to 10.12 for Aged Odds Ratio			
			0.9011 to 8.377 for Young Odds Ratio			
			(95% CI cannot be calculated for Fisher’s Exact test alone)			
*p*	Normally Distributed	2-way ANOVA	15.00 mN		−3.588 to 0.02884	
			35.00 mN		−3.934 to −0.3170	
			70.00 mN		−3.356 to 0.2608	
			140.0 mN		−1.688 to 2.081	
*q*	Normally Distributed	2-way ANOVA	15.00 mN		−3.198 to 1.455	
			35.00 mN		−3.370 to 1.284	
			70.00 mN		−3.109 to 1.544	
			140.0 mN		−2.994 to 1.691	
*r*	Normally Distributed	2-way ANOVA	15.00 mN		−1.302 to 2.701	
			35.00 mN		−0.3710 to 3.632	
			70.00 mN		2.282 to 6.286	
			140.0 mN		1.971 to 6.009	
*s*	Normally Distributed	2-way ANOVA	15.00 mN		−2.522 to 1.914	
			35.00 mN		−3.274 to 1.162	
			70.00 mN		−5.297 to −0.8607	
			140.0 mN		−5.677 to −1.170	
*t*	Non-Normally Distributed	Kruskal-Wallis Test	Aging PBS vs CFA		0.1826 to 1.322	
			Young PBS vs CFA		0.4658 to 1.511	
*u*	Non-Normally Distributed	Contingency Table followed by Fisher’s Exact Test	1.590 to 15.73 for Young Odds Ratio			
			0.5893 to 5.284 for Aged Odds Ratio			
			(95% CI cannot be calculated for Fisher’s Exact test alone)			
*v*	Non-Normally Distributed	Contingency Table followed by Fisher’s Exact Test	1.770 to 2.348 for Young 2-day CFA vs PBS Odds Ratio			
			1.597 to 1.995 for Aged 2-day CFA vs PBS Odds Ratio			
			(95% CI cannot be calculated for Fisher’s Exact test alone)			
*w*	Normally Distributed	One-way ANOVA	CFA - 8 wk vs PBS - 8 wk		0.05562 to 0.1652	
			CFA 2 days vs PBS - 2 days		−0.1246 to −0.01475	
			Aged - 8 wk PBS vs Aged 8 wk CFA		−0.06385 to 0.01858	
			Aged 2 day CFA vs Aged 2 day PBS		0.03960 to 0.1341	
*x*	Normally Distributed	One-way ANOVA	Aged 8 Wk CFA vs Aged 8 Wk PBS		−0.7127 to 0.9026	
			Aged 8 Wk CFA vs Aged 2 day CFA		−0.4550 to 1.054	
			Aged 8 Wk CFA vs Aged 2 day PBS		−0.7648 to 0.8323	
			Aged 8 Wk CFA vs Young 8 week CFA		−0.5910 to 0.9439	
			Aged 8 Wk CFA vs Young 8 week PBS		−1.376 to 0.2588	
			Aged 8 Wk CFA vs Young 2 days CFA		−0.4760 to 1.073	
			Aged 8 Wk CFA vs Young 2 days PBS		−0.6258 to 0.9712	
			Aged 8 Wk PBS vs Aged 2 day CFA		−0.6031 to 1.012	
			Aged 8 Wk PBS vs Aged 2 day PBS		−0.9101 to 0.7877	
			Aged 8 Wk PBS vs Young 8 week CFA		−0.7382 to 0.9013	
			Aged 8 Wk PBS vs Young 8 week PBS		−1.520 to 0.2131	
			Aged 8 Wk PBS vs Young 2 days CFA		−0.6228 to 1.030	
			Aged 8 Wk PBS vs Young 2 days PBS		−0.7712 to 0.9267	
			Aged 2 day CFA vs Aged 2 day PBS		−1.064 to 0.5327	
			Aged 2 day CFA vs Young 8 week CFA		−0.8905 to 0.6444	
			Aged 2 day CFA vs Young 8 week PBS		−1.676 to −0.04072	
			Aged 2 day CFA vs Young 2 days CFA		−0.7755 to 0.7735	
			Aged 2 day CFA vs Young 2 days PBS		−0.9253 to 0.6717	
			Aged 2 day PBS vs Young 8 week CFA		−0.6680 to 0.9535	
			Aged 2 day PBS vs Young 8 week PBS		−1.451 to 0.2658	
			Aged 2 day PBS vs Young 2 days CFA		−0.5526 to 1.082	
			Aged 2 day PBS vs Young 2 days PBS		−0.7013 to 0.9792	
			Young 8 week CFA vs Young 8 week PBS		−1.565 to 0.09424	
			Young 8 week CFA vs Young 2 days CFA		−0.6650 to 0.9091	
			Young 8 week CFA vs Young 2 days PBS		−0.8145 to 0.8069	
			Young 8 week PBS vs Young 2 days CFA		0.02128 to 1.693	
			Young 8 week PBS vs Young 2 days PBS		−0.1269 to 1.590	
			Young 2 days CFA vs Young 2 days PBS		−0.9432 to 0.6916	
*y*	Normally Distributed	Chi Square followed by Fisher’s exact test	1.342 to 24.34 for naive vs 8-week CFA Odds Ratio			
			1.111 to 21.82 for 2-day CFA vs 8-week CFA Odds Ratio			
			(95% CI cannot be calculated for Fisher’s Exact test alone)			
*z*	Normally Distributed	1-way ANOVA	Naive vs 2-day CFA		−31.44 to 46.29	
			Naive vs 8-week CFA		−21.96 to 85.90	
			2-day CFA vs 8-week CFA		−30.92 to 80.01	
*aa*	Normally Distributed	Chi Square	0.2197 to 31.37 for naïve vs 2-day Odds Ratio			
			0.03641 to 6.866 for 2-day vs 8-week Odds Ratio			
			0.1677 to 10.27 for naïve vs 8-week Odds Ratio			
			(95% CI cannot be calculated for Fisher’s Exact test alone)			
*bb*	Normally Distributed	1-way ANOVA	Naive vs 2-day		−99.31 to 0.3113	
			Naive vs 8-week		−90.56 to 9.061	
			2-day vs 8-week		−41.06 to 58.56	
*cc*	Normally Distributed	2-way ANOVA	Naive vs 2-day			
			Force			
			15.00		−8.125 to 1.035	
			35.00		−7.587 to 1.573	
			70.00		−7.013 to 2.147	
			140.0		−6.602 to 2.701	
			Naive vs 8 week			
			Force		−7.934 to 2.113	
			15.00		−8.314 to 1.732	
			35.00		−8.317 to 1.729	
			70.00		−7.230 to 2.816	
			140.0		−7.934 to 2.113	
			2-day vs 8 week			
			Force			
			15.00		−4.665 to 5.935	
			35.00		−5.584 to 5.017	
			70.00		−6.161 to 4.440	
			140.0		−5.619 to 5.106	
*dd*	Non-Normally Distributed	Kruskall Wallis Test		Naïve	2-day	8-week
			Lower 95% CI	0.4476	0.7617	0.9286
			Upper 95% CI	0.8724	1.083	1.929
*ee*	Normally Distributed	Student’s t-test for PBS vs CFA for each time point and gene	−0.9854 to −0.1312 Young 2-day Nav1.7			
			−1.848 to 0.07474 Young 2-day Nav1.8			
			−1.643 to 0.4202 Young 2-day Nav1.9			
			−1.886 to 0.2060 Young 2-day Piezo2			
			−2.157 to −0.7731 Young 2-day TRPA1			
			−1.294 to 0.06031 Young 2-day TRPV1			
			−1.014 to 0.1110 Young 2-day TRPC3			
			0.007134 to 1.246 Young 2-day TRPC6			
			−1.581 to 0.6142 Young 2-day Kv1.1			
			−1.399 to 0.07587 Young 2-day Kv7.2			
			−1.710 to −0.06359 Young 2-day Kv7.3			
			−0.6277 to 0.7177 Young 8-week Nav1.7			
			−0.9606 to 0.4572 Young 8-week Nav1.8			
			−0.8359 to 0.6292 Young 8-week Nav1.9			
			−1.141 to 0.8441 Young 8-week Piezo2			
			−0.7882 to 0.4815 Young 8-week TRPA1			
			−0.9529 to 0.4529 Young 8-week TRPV1			
			−1.469 to 0.8719 Young 8-week TRPC3			
			−0.5417 to 1.498 Young 8-week TRPC6			
			−0.7716 to 0.1383 Young 8-week Kv1.1			
			−1.292 to 0.4155 Young 8-week Kv7.2			
			−1.370 to 0.3438 Young 8-week Kv7.3			
			−0.6742 to 0.5142 Aged 2-day Nav1.7			
			−0.9797 to 0.9464 Aged 2-day Nav1.8			
			−0.9318 to 1.192 Aged 2-day Nav1.9			
			−0.8022 to 0.4022 Aged 2-day Piezo2			
			−2.579 to −0.4873 Aged 2-day TRPA1			
			−1.068 to 0.08780 Aged 2-day TRPV1			
			0.02838 to 0.9250 Aged 2-day TRPC3			
			−0.5024 to 1.082 Aged 2-day TRPC6			
			−0.07159 to 0.5116 Aged 2-day Kv1.1			
			−0.4090 to 0.6290 Aged 2-day Kv7.2			
			−0.4557 to 0.5357 Aged 2-day Kv7.3			
			−0.3108 to 0.5842 Aged 8-week Nav1.7			
			−0.7250 to 0.02833 Aged 8-week Nav1.8			
			−0.6878 to 0.1078 Aged 8-week Nav1.9			
			−0.7805 to 0.3605 Aged 8-week Piezo2			
			−1.981 to −0.4358 Aged 8-week TRPA1			
			−0.7546 to −0.03539 Aged 8-week TRPV1			
			−0.6783 to 0.8150 Aged 8-week TRPC3			
			−1.052 to 0.8521 Aged 8-week TRPC6			
			−0.1824 to 0.8290 Aged 8 week Kv1.1			
			−0.5183 to 0.6183 Aged 8 week Kv7.2			
			−0.4955 to 0.6255 Aged 8 week Kv7.3			
*ff*	Normally Distributed	One-way ANOVA	Nav1.7			
			Aged 2-day vs Aged 8 weeks		−0.2611 to 0.5441	
			Aged 2-day vs Young 2-day		−0.8386 to −0.03346	
			Aged 8 weeks vs Young 8 week		−0.3855 to 0.2719	
			Young 2-day vs Young 8 week		0.1920 to 0.8494	
			Nav1.8			
			Aged 2-day vs Aged 8 weeks		−1.284 to 0.7893	
			Aged 2-day vs Young 2-day		−2.104 to −0.03105	
			Aged 8 weeks vs Young 8 week		−0.7631 to 0.9298	
			Young 2-day vs Young 8 week		0.05728 to 1.750	
			Nav1.9			
			Aged 2-day vs Aged 8 weeks		−1.233 to 0.6500	
			Aged 2-day vs Young 2-day		−1.694 to 0.1896	
			Aged 8 weeks vs Young 8 week		−0.6290 to 0.9088	
			Young 2-day vs Young 8 week		−0.1685 to 1.369	
			Piezo2			
			Aged 2-day vs Aged 8 weeks		−1.071 to 1.067	
			Aged 2-day vs Young 2-day		−1.880 to 0.2578	
			Aged 8 weeks vs Young 8 week		−0.8390 to 0.9063	
			Young 2-day vs Young 8 week		−0.03008 to 1.715	
			TRPA1			
			Aged 2-day vs Aged 8 weeks		−0.8925 to 2.265	
			Aged 2-day vs Young 2-day		−1.406 to 1.752	
			Aged 8 weeks vs Young 8 week		−0.04212 to 2.536	
			Young 2-day vs Young 8 week		−0.4714 to 3.050	
			TRPV1			
			Aged 2-day vs Aged 8 weeks		−0.6561 to 0.7992	
			Aged 2-day vs Young 2-day		−0.9202 to 0.5352	
			Aged 8 weeks vs Young 8 week		−0.4682 to 0.7201	
			Young 2-day vs Young 8 week		−0.2041 to 0.9841	
			TRPC3			
			Aged 2-day vs Aged 8 weeks		−1.036 to 0.4285	
			Aged 2-day vs Young 2-day		−1.465 to −0.0002279	
			Aged 8 weeks vs Young 8 week		−0.8156 to 0.3804	
			Young 2-day vs Young 8 week		−0.3868 to 0.8092	
			TRPC6			
			Aged 2-day vs Aged 8 weeks		−0.3808 to 0.8767	
			Aged 2-day vs Young 2-day		−0.3464 to 0.9110	
			Aged 8 weeks vs Young 8 week		−0.3308 to 0.6959	
			Young 2-day vs Young 8 week		−0.3651 to 0.6616	
			Kv1.1			
			Aged 2-day vs Aged 8 weeks		−0.9476 to 1.019	
			Aged 2-day vs Young 2-day		−1.693 to 0.2736	
			Aged 8 weeks vs Young 8 week		−1.234 to 0.3716	
			Young 2-day vs Young 8 week		−0.4887 to 1.117	
			Kv7.2			
			Aged 2-day vs Aged 8 weeks		−0.9518 to 0.8372	
			Aged 2-day vs Young 2-day		−1.666 to 0.1225	
			Aged 8 weeks vs Young 8 week		−1.099 to 0.3614	
			Young 2-day vs Young 8 week		−0.3847 to 1.076	
			Kv7.3			
			Aged 2-day vs Aged 8 weeks		−1.049 to 1.010	
			Aged 2-day vs Young 2-day		−2.029 to 0.02973	
			Aged 8 weeks vs Young 8 week		−1.274 to 0.4071	
			Young 2-day vs Young 8 week		−0.2940 to 1.387	
